# On the Use of Blood-Letting in the Cold Stage of the Paroxysm of Intermittent Fever

**Published:** 1827-07

**Authors:** T. Ridgway

**Affiliations:** Licentiate of the Royal College of Physicians, London; and formerly Surgeon to the Rifle Brigade.


### THE LONDON
### Medical and Physical Journal.

N° 341, VOL. LVIII.] JULY, 1827. [N° 13, *New Series*.

For many fortunate discoveries in medicine, and for the detection of numerous errors, the world is indebted to the rapid circulation of Monthly Journals; and there never existed any work, to which the Faculty, in Europe and America, were under deeper obligations, than to the Medical and Physical Journal of London, now forming a long, but an invaluable, series.—RUSH.

### ORIGINAL PAPERS,
AND
### CASES OBTAINED FROM PUBLIC INSTITUTIONS AND OTHER AUTHENTIC SOURCES.

### INTERMITTENT FEVER.

*On the Use of Blood-letting in the Cold Stage of the Paroxysm of Intermittent Fever.* By T. RIDGWAY, M.D. Licentiate of the Royal College of Physicians, London; and formerly Surgeon to the Rifle Brigade.

DR. MACKINTOSH, of Edinburgh, has written a paper,* wherein he has distinctly and satisfactorily shown that the danger which has been apprehended from blood-letting, should it be attempted in the cold stage of the paroxysm of intermittent fever, is not real; and that this remedy may be employed as freely, and with as much safety, at this as at any other period of the disease.

He has given a high proof of his intrepidity and philanthropy, in having, in defiance of the prejudices of past ages and of the present time, demonstrated this, in the first instance in his own person, so far back as the year 1810; and he has adduced several cases, of more recent occurrence, in corroboration of the principle he has advanced.

He has also gone further, and thinks himself justified in assuming, from what he has already seen, that blood-letting may not only be resorted to with equal safety, but that it will be found to have a greater effect in the cold stage of the paroxysm than at any other period, in putting an end to or

* Inserted in the Edinburgh Med. and Surg. Journal for April 1827.

mitigating this state, and rendering it amenable to the influence of other remedies.

The retiring of the fluids from the surface of the body, and their accumulation in the central parts of the system, from whatever cause arising, during that peculiar state which constitutes the first or cold stage of the paroxysm of intermittent fever, although it must always have been noticed, had never been sufficiently insisted on as tending to serious injury or protraction of the disease. Few instances had been recorded in which the consequences were immediate and obvious: in recent cases it was usually considered as a temporary effect; a mere distention of parts capable of expansion, removeable, and generally removed, in the future stages of reaction; and even when, after a long-continued repetition of the paroxysm, a palpable effect was produced in an enlargement of the spleen, this was considered as little less than salutary, —a mere indication of the continued influence of disease, occurring in an organ of small importance to vitality, neither tending in itself to assist in the protraction of the diseased state, nor requiring the employment of measures for its own direct removal.

At a time that he was himself suffering from intermittent fever, returning in severe and successive paroxysms, uninfluenced by any remedy, Dr. Mackintosh was led to the attentive consideration of that singular series of phenomena which presents itself in this disease; and hence proceeded to draw those inferences, which, if established, may be of the utmost importance, not only in defining the treatment to be pursued in controlling the symptoms of this form of fever, but of all the other febrile affections of which it may be taken as an archetype. Reasoning from his own impressions, and the evidence of facts resting on unquestionable authority, he felt the necessity of attaching a higher degree of consequence than was usually allowed to the effects of the cold stage, which, if it was not in reality the commencement of the diseased state, was at least its first indication, preceded every paroxysm, and, by the proportion of its intensity, affected the vehemence and duration of the succeeding stages, and determined the depth of the subsequent prostration.

Of this stage, then, the principal features were the retiring of the system on itself, the shrinking of all the external parts of the body, and the concentration of the fluids that accompanies it; the result of which, even considered as a mere effect, was to be highly deprecated; but which, if assumed as a cause or precursor of the whole of the diseased state, must be carefully anticipated in its future influences.

For the purpose of averting the immediate consequences which might arise from an over-distended state of the viscera, the most obvious and direct means was to reduce the volume of the circulating fluids, and adapt their quantity to the capacity of the parts now charged with containing them.

The most favourable moment for obviating congestion was conceived to be that in which the concentration had reached its highest point. The cold stage of the paroxysm was, therefore, allowed to come on, to be fully formed, and then blood was suddenly abstracted from the venous system in sufficient quantity to answer the proposed intention.* From this bold and decisive measure resulted, not the negative effect for which it seems to have been expressly resorted to—that of merely diminishing the quantity of the circulating fluids forced to occupy a smaller space in the expansile structure of the viscera, and thereby obviating the apprehended consequences of over-distention: neither was there produced any increase of the appalling appearances that attend the state of collapse: on the contrary, no sooner was the evacuation completely effected, than the fluids rushed forward to resume their place in the superficial vessels, and the shrunken aspect of the surface vanished; the distressing feelings which belong to the cold stage were soon removed,—the subsequent stages of the paroxysm were averted,—a perfect solution of the diseased state followed, and no other paroxysm succeeded.

Led on by so very favourable, it may be said so unexpected, an issue of an experiment, which appeared fraught with danger, Dr. Mackintosh awaited an opportunity for further illustrating his principle by other examples; and, although he has not succeeded so fully in every instance, he has certainly given sufficient proof that blood-letting may be employed with perfect safety in the cold stage of the paroxsym of intermittent fever. He has also shown that, carried into effect at this period, it will often abruptly put an end to the diseased state; and that, where this does not actually happen, it will have the effect, perhaps always, of mitigating the violence of the paroxysms, and of making them susceptible of a more ready removal by other remedies; while the consequences of reneated and excessive concentration of the fluids and congestion are thereby surely obviated. It will, therefore, probably be admitted generally, that in this

* I believe that it is not often a matter of indifference whether, in the treatment of disease, blood be taken from the venous or arterial system; an opinion I have elsewhere advanced.

instance a valuable addition has been contributed to medical knowledge.

Having thus far attempted to display the existing situation of the present question, rather than to present any new object of attention, I shall now proceed to offer a few observations, which may tend to confirm the efficacy and safety of the proposed method, and also assist to determine whether that preference be just which asserts the superiority of blood-letting in the cold stage of the paroxysm, over the same remedy employed at any other period of the disease; and although I may not, perhaps from a want of sufficient experience, appear always to coincide with Dr. Mackintosh in the views he entertains on this subject, I shall hope to be understood throughout as having a proper sense of his merit.

It was at the time when the British army was in winter-quarters on the frontier of Spain, after the campaign of 1812, a period above two years later than that in which Dr. Mackintosh made the experiment in his own person, that I was forced by circumstances to employ blood-letting in the cold stage of a paroxysm of intermittent fever. Its success was complete. I was quite satisfied of its safety and efficacy; and, had I deemed it necessary in any future instance, I should have had no hesitation in resorting to it. But it will be necessary to revert to a still earlier point of time for the better understanding of the subject.

The expedition to the mouth of the Scheldt took place in 1809: the sufferings of the troops employed on that occasion are not yet forgotten. The second battalion of the Rifle Brigade was, on its return to England, sent to its former quarters at Hythe in Kent. At first almost every man was sick, and the whole of the barracks were converted into an hospital; but, as the violence of the disease abated, these returned gradually to their original use and designation. The soldiers, however, still continued for some time to be little better than convalescent, and hardly capable of performing their usual active duties. When the battalion was in this state, early in the year 1810, I was waited on one morning by a corporal, whose duty it was to announce such matters, and informed that a man was dying in his barrack-room. I instantly went with him, and saw that the man was really in those convulsions which often immediately precede death; and he was no sooner removed into the hospital than he actually expired. Again, another morning the same thing happened; and this for three or four times. Upon strict inquiry among the soldiers, I found out, what they were not at first disposed to avow, and from the state of the battalion it had not been difficult to conceal, that these men had severally been affected with a paroxysm of intermittent fever of no great violence, twice before, at tertian periods; and that this was, in fact, the accession of the third paroxysm which had terminated their existence. The symptoms which preceded death had been such as to impress me with the belief that the immediate cause thereof existed in the head; and, by examination, I satisfied myself that the brain had been incapacitated from performing its functions in consequence of pressure, from the repression or retiring back, as it might be, of the fluids into the cavity of the skull, during the effort to establish the first stage of the approaching paroxysm.

Adopting the line of reasoning which appears in the former part of this paper, (and it would be useless to recapitulate,) I resolved to diminish the volume of the fluids, that they might the more readily accommodate themselves to the space which, on the supervention of the cold stage, they must occupy. To await the formation of the paroxysm, and the presence of the fatal symptoms I had witnessed, appeared to be imprudent; I therefore at once determined to anticipate the paroxysm, if possible, and avert the impending danger. The non-commissioned officers were charged to detect and expose intermittent fever in its first approach; the period of accession was noted, and, before the next paroxysm could enter on its formation, so much blood was removed from the venous system as seemed sufficient to meet the effect of the approaching concentration. It would, perhaps, be considered gratuitous to assume that, by these means, the paroxysm was in any instance averted; and, indeed, it is not intended to detail minutely events which happened so long ago, and under such peculiar circumstances of difficulty and distress. Suffice it then to say, that henceforward no fatal event arose from this cause; that the paroxysms, when they were continued, were rendered uniformly mild and tractable, and to be interrupted with perfect facility by other remedies; while congestion, as a consequence of their repetition, was of course unknown, and the re-establishment of the general health was effected more rapidly than by any other means, and was complete and permanent. It is not then to be wondered at that this method of treatment continued to precede all others in my estimation, and became established in my practice.

Early in the year 1813, while the army was yet in winter quarters on the frontier of Spain, a soldier was received into the regimental hospital of the third battalion Rifle Brigade, who was first seen in the second paroxysm of a tertian intermittent, so fully formed as to afford no hope of its interruption. According to custom, the period of accession was marked, for the purpose of noting the time at which blood-letting was to be performed, so that it might immediately precede the following paroxysm. About an hour, then, as near as could be calculated, before this was expected to show itself, I presented myself at the hospital, but found that the paroxysm had anticipated its former period considerably, and that the cold stage was now distinctly formed. Disappointed at this, and anxious to save the man from a third paroxysm, as violent in all probability as the former, I determined to proceed, regardless of this unexpected obstacle, and took from his arm about twelve ounces of blood. To my utter astonishment, the cold, and other stages of the paroxysm, glided away imperceptibly; the disease was gone; and, after awaiting the next period of accession, the soldier returned to his duty, and never after made his appearance at the hospital.

In this manner was forced on me, as it were, the assurance that the presence of the cold stage was no impediment to the employment of blood-letting; and thus did I become conscious, not only of its safety, but of its efficacy in this stage of the paroxysm: but this instance was not alone sufficient to impress me with its superiority over the method by anticipation, which I had before employed; although I may confess that neither opportunity has offered, or inclination prompted me, to make a comparative trial of their merits. In the hot stage, blood-letting has been employed, as it would appear, with little benefit: it may possibly be not altogether devoid of an injurious effect, in repressing the reaction. In the sweating stage, even should it have no injurious tendency, it would seem to be useless and unnecessary,—at least as far as the existing paroxysm is concerned. In the interval, or period of inaction, it is probable that the diseased state, in whatever it may consist, still exists, although its movements may be less perceptible: in short, that there is an apparent, but no real interval. The first part of this stage is characterised by excessive depression, resulting from the preceding violent agitations of every part of the system. As long as this sense of extreme weakness continues, blood-letting would appear to be altogether inadmissible; and truly there can hardly be conceived any reason for its employment. In the latter part of the interval, when the system has risen out of this state of overwhelming depression, and is preparing to describe anew her circle of active operation, this remedy may be resorted to with perfect confidence.

It has been shown by Dr. Mackintosh, and admitted and corroborated by myself, that blood-letting may be employed in the cold stage of the paroxysm with safety and great advantage: between this stage, then, and that portion of the interval which immediately precedes it, appears to me to rest at present the question of preference, which experience has not yet sufficiently determined. The distressed feelings of the sufferer, the appalling appearances of shrinking and debility, and the difficulty of taking away a sufficient quantity of blood, will be obstacles to the use of blood-letting in the cold stage, although not such as to counterbalance important benefits. In the malignant and fatal forms of intermittent, in which the accession of the cold stage is the signal for the destruction of the patient, it would seem to be more prudent to anticipate than to await the presence of those symptoms which bring death in their train, and which it is probable the remedy would not then have the power of averting.

As far as my own limited sphere of observation has extended, and enabled me to judge, blood-letting is a remedy of the first importance in intermittent fever, employed with judgment and moderation; and this not only in its earlier periods, but when the disease has become protracted to a considerable length, and visceral congestion has completely established itself.

Where the paroxysms of intermittent fever proceed, notwithstanding the employment of blood-letting and its concomitant remedies, or where they have appeared to be in themselves of a mild and tractable nature, I have seen no remedy so effectual in interrupting their progress as the Peruvian bark; but I confess that I have sometimes had reason for deep regret, in having trusted to this as the principal remedy. In a former number of this Journal,* I showed that the best method of giving the Peruvian bark was in one large and sufficient dose, immediately before the formation of the paroxysm, instead of the usual mode of exhibiting it in a number of lesser doses, distributed throughout a portion of the interval. Having been on the Peninsula since that period, I had an opportunity of ascertaining that the practice of giving the large undivided quantity was that which was in use among the empirics of Spain. It is probable, therefore, that this was the original method, and that the other was a refinement of medicine and modern times. At the time that I wrote the paper on this subject, it was not known that the chemical preparations of the bark might be administered with

* For April 1825.

safety in considerable quantity: lately, however, this has been ascertained, and they have been applied on this principle to anticipate the approaching paroxysm.

In a former part of this paper I ventured to remark, that the principle displayed and advocated by Dr. Mackintosh, of bleeding in the cold stage of the paroxysm of intermittent, might possibly be found applicable in other forms of fever; and I think I have observed that, when blood-letting has been employed in continued fever, before the disease has had time to be fully developed, it has been much more effectual than at a later period. I shall, however, relate one case which appears to bear on this point.

A soldier of the third battalion Rifle Brigade was received into the Regimental Hospital at Dublin, in 1818, with continued fever. He was not more than eighteen years of age, strong, robust, full and florid in his appearance, and, being a musician, but little subject to the common causes of disease in soldiers. The symptoms were slight, and, seeming to require no very powerful remedy, a dose of neutral salt, and the regimen of the hospital, was trusted to at the moment for its removal. Next morning, on entering the ward, I was struck with horror at the change in his appearance: he lay unmoved upon his back; the plumpness and freshness of his youthful countenance were exchanged for the aspect of death; his eyes, kept fixed steadily on me, were sunk in their orbits, which looked like two hollow cups in deep shadow; the face was shrunk, and of a livid hue. Remembering how often I had been disappointed in the use of stimulants, which seemed to hurry rather than retard a threatened fatal termination, I determined in this instance to try the effect of blood-letting, emboldened by that which it had produced in the cold stage of intermittent. There was no tremor or agitation of the body, no uneasiness, but an unusual tranquility; the temperature of the surface was not high, and the pulse at the wrist was small and very frequent, (170 in a minute.) I opened a vein; the blood flowed slowly. I kept my finger on the artery, but it did not falter; neither were its strokes altered in frequency or strength. Having succeeded in obtaining about eight ounces, I thought the experiment had been carried far enough for the time, and waited the event. After some hours, little alteration had taken place; but the next morning the hideous aspect of the countenance was evidently diminished, and its plumpness and freshness seemed to be returning gradually. I then, without fear, took other eight ounces of blood, which was soon followed by a perfect restoration of health and appearance; so that, without any other remedy, he was able in a few days to resume his usual avocations.

In the Number of this Journal for October 1824, is an extract of a letter from Mr. Sardham, surgeon to the forces, serving, I presume, in the East Indies, which contains some observations that appear to me to be not irrelevant to this subject. He speaks of cholera. "Nine well-marked cases presented themselves in May and June, the two first of which terminated fatally, and the morbid appearances in them have produced a very considerable change in my ideas as to the nature of the disease, and I believe enabled me to save the rest. I have been impressed with the opinion that it is in consequence of irregular or suspended action of the brain all the other symptoms arise; the circulation is almost stopped; the blood retires from the surface; the heart is incapable of its office; congestion takes place in the head, and particularly in the liver." * * * "When I first had to encounter this disease, I objected to, and did not use, the lancet, because I knew inflammation of the stomach and intestines to be a consequence, and not a cause; for in all those in whom it was most acute, and terminated fatally in a few hours, neither the stomach nor intestines ever showed a red vessel, but there was the greatest venous congestion. But, since the change in my ideas relative to the nature of the disease, I have, where I could get it, immediately bled to the extent of from forty to even sixty ounces, and with the most marked good effects; the patients finding great and immediate relief, the heart resuming its office, the pulse returning to the wrist, the surface losing its shrunken livid appearance and deadly coldness, and the cramps, vomiting, and implacable thirst being greatly moderated."

Whatever, then, may be the immediate occasion of this effort at concentration, which forms so prominent a feature of certain diseased states, it is evident that, by the removal of a portion of the general mass of blood, the crassamentum with the serum, the process on which it depends seems to be most effectually subverted, and the necessity for reaction removed or diminished : while it also becomes perfectly obvious that the risk supposed to attend this measure in such situations is not real, but imaginary.

*London; May 6th*, 1827.

